# Mechanical Ventilation Lessons Learned From Alveolar Micromechanics

**DOI:** 10.3389/fphys.2020.00233

**Published:** 2020-03-24

**Authors:** Michaela Kollisch-Singule, Joshua Satalin, Sarah J. Blair, Penny L. Andrews, Louis A. Gatto, Gary F. Nieman, Nader M. Habashi

**Affiliations:** ^1^Department of Pediatric Surgery, Arkansas Children’s Hospital, Little Rock, AR, United States; ^2^Department of Surgery, SUNY Upstate Medical University, Syracuse, NY, United States; ^3^Department of Critical Care, R Adams Cowley Shock Trauma Center, University of Maryland Medical Center, Baltimore, MD, United States; ^4^Department of Biological Sciences, SUNY Cortland, Cortland, NY, United States

**Keywords:** alveolar stability, alveolar heterogeneity, lung injury, *in vivo* microscopy, micromechanics

## Abstract

Morbidity and mortality associated with lung injury remains disappointingly unchanged over the last two decades, in part due to the current reliance on lung macro-parameters set on the ventilator instead of considering the micro-environment and the response of the alveoli and alveolar ducts to ventilator adjustments. The response of alveoli and alveolar ducts to mechanical ventilation modes cannot be predicted with current bedside methods of assessment including lung compliance, oxygenation, and pressure-volume curves. Alveolar tidal volumes (Vt) are less determined by the Vt set on the mechanical ventilator and more dependent on the number of recruited alveoli available to accommodate that Vt and their heterogeneous mechanical properties, such that high lung Vt can lead to a low alveolar Vt and low Vt can lead to high alveolar Vt. The degree of alveolar heterogeneity that exists cannot be predicted based on lung calculations that average the individual alveolar Vt and compliance. Finally, the importance of time in promoting alveolar stability, specifically the inspiratory and expiratory times set on the ventilator, are currently under-appreciated. In order to improve outcomes related to lung injury, the respiratory physiology of the individual patient, specifically at the level of the alveolus, must be targeted. With experimental data, this review highlights some of the known mechanical ventilation adjustments that are helpful or harmful at the level of the alveolus.

## Introduction

Acute lung injury is caused by a pathologic tetrad of alveolar instability, endothelial leakage, alveolar edema, and surfactant dysfunction (Nieman et al., 2018). Atelectrauma and associated repetitive opening and collapse of the injured alveoli results in alveolar instability (Schiller et al., 2001). An increase in pulmonary capillary permeability (Matthay et al., 2012) combined with damage to the alveolar-capillary barrier leads to alveolar edema (Budinger and Sznajder, 2006). This edema and repetitive opening and collapse of unstable alveoli alters surfactant function and abundance, resulting in increased surface tension (Greene et al., 1999; Warriner et al., 2002), and promotion of alveolar epithelial injury (Ruhl et al., 2019). Finally, tissue injury activates pro-inflammatory signaling, leading to biotrauma (Tremblay et al., 1997). Although mechanical ventilation can be necessary to support the injured lung in order to promote gas exchange, it can itself be injurious by exacerbating atelectrauma and recruitment/derecruitment ultimately leading to the acute respiratory distress syndrome (ARDS) (Gajic et al., 2004; Albert, 2012).

Despite decades of research into protective mechanical ventilation strategies, mortality rates associated with ARDS have remained disappointing with mortality rates ranging from 34.9 to 46.1% (Bellani et al., 2016) despite the use of protective non-injurious ventilation (Villar et al., 2011). When a patient is placed on the mechanical ventilator, the ventilator settings are modified to accommodate the physiology of the lung [tidal volumes (Vt), compliance, pressure-volume curves, oxygen saturation, etc…], while taking into account the patient’s underlying volume status and cardiac physiology (Vieillard-Baron et al., 2016). Regional microscale responses [in the alveoli, conducting airway, and interstitium] are not predictable from the macroscale inputs of the mechanical ventilator (Kollisch-Singule et al., 2014a). Therefore, the universal application of generic mechanical ventilator settings to all patients without considering patient-specific differences in the underlying micromechanics may not benefit the lung and even cause further injury. Personalizing mechanical ventilation in order to link the macro-parameters set on the mechanical ventilator to the dynamic changes that occur at the level of the alveolus will be important to attenuate the pathophysiology of lung injury: alveolar instability, endothelial leakage, alveolar edema, and surfactant dysfunction (Kollisch-Singule et al., 2018; Nieman et al., 2018). Herein, we review the current literature investigating the response of the lung micro-anatomy: the alveoli and alveolar ducts, to varying mechanical ventilation settings.

## Alveolar Recruitment

The majority of what is known about alveolar micromechanics is based on *in vivo* and *in vitro* models (Tschumperlin et al., 2000; Perlman and Bhattacharya, 2007; Mertens et al., 2009) incorporating methods of histology, electron microscopy, *in vivo* microscopy, optical coherence tomography, computed tomography, synchrotron imaging, electrical impedance tomography, inhaled polarized gases, and computer-based theoretical models (Broche et al., 2017; Grune et al., 2019), but there are no direct methods of assessing alveolar micromechanics, particularly the response to mechanical ventilation strategies (Roan and Waters, 2011).

At the level of the lung, it is well-recognized that even relatively high pressures are not harmful to the lung when applied tonically (as static strain) but becomes harmful when applied dynamically (as dynamic strain or with large driving pressures) (Protti et al., 2013; Amato et al., 2015; Jain et al., 2017). This does correlate with alveoli in that cyclic stretching and higher amplitude deformation of alveolar epithelial cells leads to greater cell damage whereas static or lower amplitude deformations do not (Tschumperlin et al., 2000). An individual alveolus requires a certain pressure to re-open (critical opening pressure) and also have a pressure where it will collapse (critical closing pressure) (Albert et al., 2009) which is less than the opening pressure. This suggests that, in order for a recruited alveolus to remain open, the system must have a certain minimum airway pressure that is greater than the alveolar closing pressure, but does not need to be as high as the opening pressure (Bates and Irvin, 2002). Therefore, the application of positive end expiratory pressure (PEEP) at or above the alveolar closing pressure offers a static level of pressure to prevent alveolar derecruitment (Bates and Irvin, 2002).

*In vivo* microscopy has been used to directly monitor the alveolar recruitment patterns in response to lung injury and varying levels of PEEP (Kollisch-Singule et al., 2014a, 2016). Such studies have demonstrated that higher levels of PEEP lead to increased alveolar recruitment and surface area with particular benefit derived from mechanical ventilation strategies that offer a higher mean airway pressure (Paw) (Kollisch-Singule et al., 2014a), which allows for improved intratidal recruitment without causing alveolar overdistension (Smith et al., 2015). The distension that occurs at the level of the alveolus and alveolar duct in response to the stress of the mechanical ventilator is the micro-strain and has been calculated as:

microstrain=ΔL/PLPe

where ΔL_P_ is the change in perimeter length between inspiration and expiration and L_Pe_ is the original perimeter length at expiration (Kollisch-Singule et al., 2014a). Using this calculation, it was shown that alveoli and alveolar ducts are both exposed to less micro-strain with higher levels of PEEP and P_aw_ (Kollisch-Singule et al., 2014a, b).

Although PEEP is critical to stabilizing both the alveoli and alveolar ducts (Kollisch-Singule et al., 2014a, b), it may cause impaired minute ventilation and subsequent hypercapnia (Acute Respiratory Distress Syndrome Network et al., 2000), and also may lead to a point when PEEP increases do not lead to any additional stability. This is the so-called ‘best PEEP,’ however there is no consensus over what ‘best’ PEEP is. The ‘best PEEP’ should be dependent on the underlying alveolar compliance, alveolar closing pressures, and time (Bates and Irvin, 2002; Albert et al., 2009), but the majority of PEEP titration strategies do not provide a micromechanical basis (Chiumello et al., 2014; Pintado et al., 2017; Writing Group for the Alveolar Recruitment for Acute Respiratory Distress Syndrome Trial Investigators et al., 2017; Beitler et al., 2019). Alveolar stabilization may occur at varying levels of PEEP depending on the degree and type of underlying injury (Carvalho et al., 2008). In one murine model of direct lung injury, the ‘best PEEP’ leading to alveolar stability was found to be 9 cmH2O (Andrews et al., 2015), whereas in another it was at 16 cmH2O (Kollisch-Singule et al., 2014a). In a rabbit model of lung injury utilizing synchrotron imaging to estimate the response of the terminal airway to varying levels of PEEP, Broche et al. (2017) found that increasing PEEP led to improved lung aeration and that terminal airway derecruitment increased markedly for PEEP less than 6 cmH_2_O. The study also revealed that, although recruitment/derecruitment was attenuated with increasing PEEP, it was still present at PEEP levels as high as 12 cmH2O (Broche et al., 2017). Although creative methods have been used to determine the best PEEP of the lung using bedside measurements, these strategies do not account for the variability or physiology of the alveoli and alveolar ducts (Chiumello et al., 2014; Pintado et al., 2017; Writing Group for the Alveolar Recruitment for Acute Respiratory Distress Syndrome Trial Investigators et al., 2017; Beitler et al., 2019). For instance, using *in vivo* microscopy, alveolar recruitment and derecruitment were compared against the inflection points of the pressure-volume curves and overall found to have a poor correlation, with the exception of the deflation limb upper inflection point, which was found to be related to the derecruitment pressure (Dirocco et al., 2007).

In a porcine model of acute lung injury, a recruitment maneuver was performed followed by stepwise decreases in PEEP with CT scans at end-inspiration and end-expiration, as well as calculation of respiratory system elastance, stress index, and percentage of volume-dependent elastance (Carvalho et al., 2008). The method of injury (pulmonary injury by lavage versus extrapulmonary lung injury with systemically administered oleic acid) changed the pulmonary mechanics such that the extrapulmonary lung injury revealed greater stability at higher PEEP (Carvalho et al., 2008), consistent with prior studies comparing the etiology of lung injury to PEEP-responsiveness (Gattinoni et al., 1998). The stress index and percentage of volume-dependent elastance were meant to be used as surrogates for alveolar hyperinflation but were found to be useful only in the non-injured lungs, as there was no significant change to either when PEEP was reduced in the injured lungs (Carvalho et al., 2008). In the injured lung, recruitment and overdistension were found to occur simultaneously, with higher PEEP leading to recruitment of dependent lung regions but hyperinflation of non-dependent regions (Carvalho et al., 2008). The study determined that the PEEP that generated the lowest respiratory system elastance provided the best balance between lung recruitment and hyperinflation (Carvalho et al., 2008). But the study also served to demonstrate that strict reliance on pressure-volume curves may fail in the injured lung when there is co-existence of hyperinflation and collapse (Carvalho et al., 2008).

Oxygenation has also been found to be a poor marker of alveolar stability (Cereda et al., 2016b). In a murine model of direct lung injury and ventilator induced lung injury, strain (but not oxygenation) was found to predict propagation of lung injury (Cereda et al., 2016b). In a murine model of lung injury, alveolar stability was achieved at 9 cmH_2_O with further increases in PEEP not leading to an increase in microscopic stability despite an improvement in oxygenation (Andrews et al., 2015). In another *in vivo* study, increases in PEEP from 5 to 10 cmH_2_O led to only a transient increase in oxygenation despite a marked improvement in stability with increasing PEEP (Halter et al., 2003). This has been demonstrated in other studies in which repeated recruitment maneuvers are necessary to maintain oxygen saturation (Fujino et al., 2001) with higher PEEP at the conclusion of the recruitment maneuver being crucial to maintaining open alveoli and sustained improvements in oxygenation (Rimensberger et al., 1999).

## Alveolar Tidal Volume

The low tidal volume (LVt) ventilation strategy has been embraced as a standard method of minimizing lung injury in patients with ARDS (Acute Respiratory Distress Syndrome Network et al., 2000), yet LVt has not demonstrated a persistent reduction in ARDS-associated mortality (Meade et al., 2008; Villar et al., 2011). This is likely because this strategy does not account for variability in patient disease, individual pulmonary mechanics, nor the alveolar micro-environment (Kollisch-Singule et al., 2018). A subsequent retrospective analysis of the LVt data (Acute Respiratory Distress Syndrome Network et al., 2000) revealed that the change in mortality was due to differences in driving pressure, or the pressure differential between inspiration and expiration (Amato et al., 2015). This study therefore demonstrated that when ventilator settings are adjusted to the lung compliance, an individual patient measurement, lung injury as measured by mortality may be attenuated (Amato et al., 2015). Even LVt can generate altered stress distribution in diseased state conditions such as emphysema (De Ryk et al., 2007) whereby stress concentrates in the injured alveoli, both at the perimeter of the weakened regions as well as the septal junction points (De Ryk et al., 2007).

LVt ventilation strategies in and of themselves do not lead to alveolar stabilization where alveolar stability is assessed by low *alveolar* volume change (Schiller et al., 2001; Kollisch-Singule et al., 2014a). The number of open and homogenous alveoli upon which a Vt is distributed is a greater determinant of alveolar stability and resulting alveolar Vt than the Vt set at the level of the mechanical ventilator (Kollisch-Singule et al., 2014a, 2016). Thus, factors that increase the number and openness of alveoli, such as increased P_aw_, lead to alveolar stabilization (Halter et al., 2003).

In a murine model of direct lung injury, LVt (6 mL⋅kg^–1^) was set at the level of the mechanical ventilator but the alveolar Vt (dynamic change in alveolar cross-sectional area between inspiration and expiration) varied considerably (Kollisch-Singule et al., 2014a). The alveolar Vt was less dependent on the set Vt on the ventilator but more on the Paw (Kollisch-Singule et al., 2014a). High alveolar Vt was observed when P_aw_ was low, even though the lung Vt was held constant at 6 mL⋅kg−^1^ (Kollisch-Singule et al., 2014a). Alveolar Vt and micro-strain decreased only once PEEP was set at 10 cmH_2_O or greater, as the associated alveolar recruitment allowed for additional surface area for the same Vt to be distributed across evenly (Kollisch-Singule et al., 2014a). This study demonstrated that even the LVt strategy may be deleterious when not applied to an open lung, but also revealed that higher Vt of up to 11 mL⋅kg^–1^ set on the ventilator can be associated with low alveolar Vt and micro-strain when combined with higher P_aw_ and a concomitant increase in alveolar recruitment (Kollisch-Singule et al., 2014a).

These findings are supported by a confocal study by Namati et al. (2008) in which alveolar size was found to increase as airway pressure increases, with a stabilization in alveolar number at ∼25 cmH_2_O. Following this, subsequent increases in pressure above 30 cmH_2_O led not to additional alveolar strain, but to alveolar recruitment, with an increase in alveolar number but a decrease in alveolar size as measured by chord length (Namati et al., 2008). One hypothesis for this is that a separate population of daughter alveoli are recruited during times of increased pressure in order to re-distribute stress amongst the alveoli (Namati et al., 2008). In another alveolar study in which alveoli were exposed to inflation pressures as high as 40 cmH_2_O, the alveoli were not observed to overdistend but rather to increase in number (Dirocco et al., 2007). This data must be interpreted with caution as 40 cmH_2_O may be a supraphysiologic pressure for a mouse (Zosky et al., 2008), however, pressures in excess of 40 cmH_2_O have not demonstrated structural lung parenchymal damage (Soutiere and Mitzner, 2004), and the lung mechanics ultimately returned to baseline, even after being stressed to 40 cmH2O (Zosky et al., 2008). Zosky et al. (2008) found that mice have a standard sigmoid pressure-volume curve up to 20 cmH_2_O but that subsequent inflation to 40 cmH_2_O led to a significant elevation in the slope of the pressure-volume curve, and a double sigmoidal pattern. One possibility for this finding is that there is a second population of alveoli that are not recruited until a pressure above 20 cmH_2_O is reached (Soutiere and Mitzner, 2004) but begin to recruit at higher pressures, consistent with the findings of Dirocco et al. (2007) and Namati et al. (2008). This newly recruited second population of alveoli then inflate smoothly during subsequent inhalations, leading to an overall improvement in compliance and an increase in tidal volume (Zosky et al., 2008). The findings of these studies demonstrating more alveolar recruitment and less overdistension were corroborated by a large animal model in which the majority of lung volume change was secondary to recruitment/derecruitment, but not due to alveolar distension (Carney et al., 1999).

## Alveolar Recruitment Over Time

The other component to mechanical ventilation that is seldom discussed but remains of critical importance for alveolar micromechanics is that of time. Alveolar opening and closing pressures are in part determined by both time and lung volume history (Bates and Irvin, 2002), as well as the open/closed status of the neighboring alveoli (Broche et al., 2017). In an *in vivo* study, alveolar recruitment was monitored over a 40 s recruitment maneuver (Albert et al., 2009). Despite keeping the recruitment pressure static, alveoli continued to recruit over the 40 s duration, suggesting that not only pressure but the time over which the pressure is held is vital to both alveolar recruitment and stability (Albert et al., 2009). Recruitment maneuvers, or pressure spurts over a short of time, in the absence of a sufficient PEEP allows for potential collapse of the alveoli that had just been recruited (Halter et al., 2003). In another *in vivo* study of alveolar responses to mechanical ventilation, brief increases in pressure with Vt failed to induce sustained alveolar recruitment, supporting the importance of the inspiratory time on alveolar opening (Kollisch-Singule et al., 2014a). Although medium to high PEEP attenuates some degree of alveolar collapse at end expiration, extended expiratory times led to alveolar derecruitment (Kollisch-Singule et al., 2014a). Taken together, the inspiratory to expiratory ratio plays an important role in alveolar stability with brief inspiratory times and extended expiratory times leading to increased alveolar micro-strain with wide amplitude swings between inspiration and expiration (Kollisch-Singule et al., 2014a).

The development of alveolar instability is not immediate and is dependent on the time alveoli are exposed to injurious mechanical ventilation settings (Pavone L.A. et al., 2007). In a murine model, alveoli that are initially exposed to injurious mechanical ventilation settings in the form of low PEEP with a high driving pressure did not immediately reveal instability (Pavone L.A. et al., 2007). Progressive injury, measured in the form of recruitment/derecruitment, took up to 30 min to manifest, with progressive injury demonstrated over time (Pavone L.A. et al., 2007).

The importance of time is not limited to the alveoli. The alveolar ducts in the setting of low PEEP with an extended expiratory time and brief inspiratory time can be injuriously distended with increased micro-strain (Kollisch-Singule et al., 2014b). Alveolar collapse in proximity to an airway can lead to heterogeneous parenchymal tethering which can impact airway stability and peribronchial stress (Ryans et al., 2019). This parenchymal heterogeneity can drive further injury, however, higher mean airway pressures, a function of both pressure and time, also have a stabilizing effect on the parenchyma (Roy et al., 2013; Ryans et al., 2019).

Yamaguchi et al. (2017) demonstrated in an *in vitro* model of non-uniform bifurcating airways that flow will divert to the airways of lower resistance. Variation in airway width affects both hydraulic and capillary pressures and therefore has a greater divergence pattern as compared with variations in airway length, which affects hydraulic resistance alone (Yamaguchi et al., 2017). The study demonstrated that a 15% change in width between two airways can lead to a 100-fold change in relative velocity through the airways when combined with high surface tension, but that lower surface tension has a protective effect on asymmetric reopening (Yamaguchi et al., 2017).

In a mathematical model of a heterogeneous airway network, Stewart and Jensen (2015) found that the air finger, which mimics the pressure delivered by the mechanical ventilator, halts its progression through the airway network at specific points. These points of stagnation occur where the ductal opening pressure is greater than the delivery pressure, and the airway network will not open until either the airway pressure or the time of this applied pressure is increased, which triggers further recruitment (Stewart and Jensen, 2015). Full recruitment of the airway network was found to be dependent on the airway pressure, the time a given pressure was applied, and airway heterogeneity (Stewart and Jensen, 2015).

## Alveolar Edema

One of the primary physiologic factors leading to ARDS is the accumulation of alveolar edema, which impairs gas exchange, deactivates surfactant, and leads to heterogeneous mechanical ventilation and ultimately a higher mortality (Greene et al., 1999; Warriner et al., 2002). Stress is concentrated between healthy and edematous alveoli (Wu et al., 2017). This stress level is proportional to the surface tension and can be exacerbated by mechanical ventilation (Wu et al., 2017).

**FIGURE 1:**
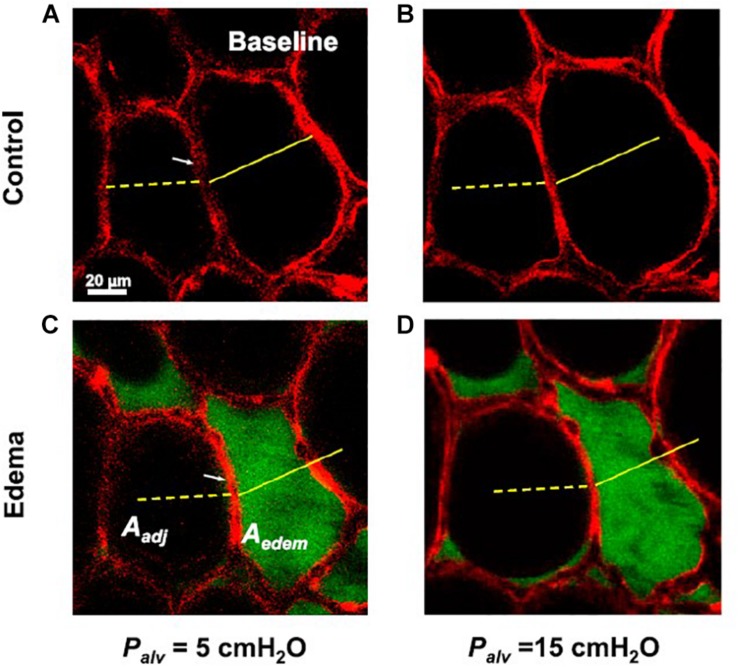
Confocal microscopy is utilized to determine the effect a single liquid-filled alveolus has on a neighboring alveolus with changes in PEEP (black: air, red: alveolar epithelium). **(A)** The alveolar diameters at baseline at a PEEP of 5 cmH_2_O **(B)** increase with a PEEP increase to 15 cmH_2_O. **(C)** Introduction of edema (green: albumin) to the alveolus leads to a decrease in diameter of the liquid-filled alveolus but alters the trans-septal pressures such that the neighboring alveolus increases in diameter to accommodate. **(D)** The fluid-filled alveolus maintains its ability to expand with PEEP increases, suggesting the individual alveolar compliance is unchanged, whereas the neighboring alveolus loses compliance (Perlman et al., 2011). *Published with permission*.

In a study by Perlman et al. (2011) confocal microscopy was utilized to determine the effect of a single liquid-filled alveolus on neighboring alveoli when exposed to varying pressures. In that study, the edematous alveolus decreased in size at a PEEP of 5 cmH_2_O but maintained its ability to increase in size in response to rising pressure, suggesting the individual alveolar compliance is unchanged (Perlman et al., 2011). But the presence of edema changed the *trans*-septal pressures such that the septum bowed toward the fluid-filled alveolus, mandating that the neighboring alveoli increased in size to accommodate, with a concomitant loss in compliance by approximately 24% (Figure 1) (Perlman et al., 2011). This phenomenon is well-supported by simulation models of injured and healthy alveoli which reveal that the recruitment maneuvers and pressures necessary to stabilize injured alveoli may lead to overdistension of the neighboring alveoli (Schirrmann et al., 2010).

Alveolar edema is also heterogeneous with alveolar flooding of the dependent regions of the lung and less flooding of the non-dependent regions (Wu et al., 2014). In an isolated perfused lung model with induced alveolar flooding, the permeability of the alveolar-capillary barrier increased over the study duration (Wu et al., 2014). Ventilation in all forms was found to be injurious, particularly with higher tidal volume, PEEP, and surface tension (Wu et al., 2014). This study therefore highlights the importance of protecting the alveoli and alveolar ducts from edema with protective mechanical ventilation before the injury has a chance to occur (Nieman et al., 2018).

In another isolated perfusion lung model of both local and global alveolar edema, the impact of surface tension and accelerated deflation on alveolar edema clearance was studied (Wu et al., 2017). In lungs with local edema alone, and thus a relatively normal surface tension, accelerated deflation led to release of fluid from flooded alveoli, allowing for redistribution of the fluid, and a decrease in flooding heterogeneity (Wu et al., 2017). In lungs with global edema, accelerated deflation also led to release of fluid from flooded alveoli, but the fluid redistributed to neighboring alveoli due to the higher surface tension, with no improvement in flooding heterogeneity (Wu et al., 2017). The importance of accelerated deflation on mucous clearance has similarly been described in a porcine lung model and represents a ventilator setting that may alter the lung micro-environment (Mahajan et al., 2019).

## Alveolar Heterogeneity

An injured lung induces heterogeneity at the level of the terminal airways which concentrates regional stress secondary to atelectasis, edema, and alterations in alveolar surface tension (Greene et al., 1999; Mertens et al., 2009; Schirrmann et al., 2010). This regional stress can concentrate alveolar strain to up to quadruple that of global strain (Rausch et al., 2011), propagating additional alveolar injury, apoptosis (Tschumperlin and Margulies, 1998), inflammation (Albaiceta and Blanch, 2011), and even membrane rupture (Vlahakis and Hubmayr, 2005). Given the degree of heterogeneity that manifests both at the regional and at the microscopic level in the injured lung, a given Vt will not distribute evenly but will rather preferentially distribute to the regions of increased alveolar compliance (Cressoni et al., 2014; Zhao et al., 2014).

**FIGURE 2:**
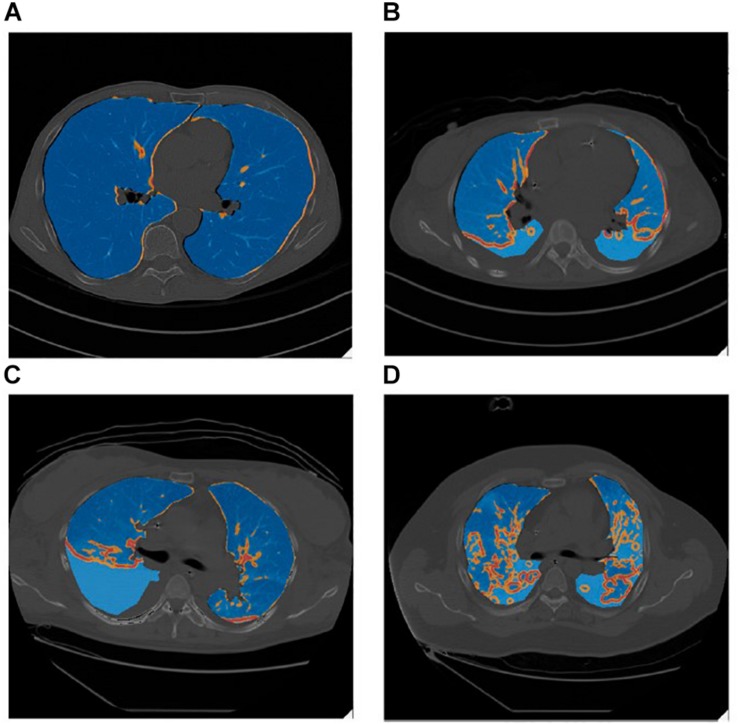
Lung inhomogeneity can be visualized grossly on CT scan as evident in these false-color images in which stress-raisers are color mapped (blue = regions of mild stress-raisers, orange = regions of moderate stress-raisers, red = regions of severe stress-raisers). Blue intensity differences are due to overlapping between the underlying CT scanning images and the false-color map of lung inhomogeneities. **(A)** Represents a control subject with healthy lungs taken at end-inspiration. **(B)** a patient with mild ARDS, **(C)** a patient with moderate ARDS, **(D)** a patient with severe ARDS, all taken during the expiratory phase with a PEEP of 5 cmH_2_O. The regions of stress-risers expose the neighboring alveoli to nearly double the applied transpulmonary pressure (Cressoni et al., 2014). *Published with permission.*

Lung inhomogeneity is correlated with not only the severity of ARDS but also with patient mortality (Cressoni et al., 2014). The degree of lung inhomogeneity in a patient with ARDS can be quantified using computerized tomography scans and determining regions of stress-risers where there is an inferred regional amplification of forces (Figure 2) (Cressoni et al., 2014). Theoretically, when lungs are healthy and homogenously inflated, a delivered pressure will distribute across an open set of alveoli and the load-bearing elements will be exposed to the same degree of stress, such that the stretch elements will uniformly expand (Roan and Waters, 2011). In reality, stress is concentrated in regions of inhomogeneity where an open alveolus is adjacent to a collapsed or fluid-filled alveolus (Mead et al., 1970). Cressoni et al. (2014) calculated this stress multiplication factor to be an average of 1.9, concluding that these regions of increased stress are exposed to nearly double the applied transpulmonary pressure (Figure 2).

For the majority of patients, lung inhomogeneity decreases with increasing airway pressures but this is not true in all patients in which PEEP, lung inhomogeneity, and lung recruitability were not related (Cressoni et al., 2014). This is consistent with other studies which have demonstrated that the best PEEP is not always the highest PEEP (Kollisch-Singule et al., 2014a), particularly when patients are stratified according to whether the ARDS is pulmonary or extrapulmonary in origin (Gattinoni et al., 1998), and has prompted searches for an algorithm to calculate the best PEEP for an individual patient (Chiumello et al., 2014). These scaled PEEP maneuvers, however, do not consider the heterogeneity within the subunits of the lung where regional specific tissue elastance, edema, and atelectasis will have different responses to the macro-parameter changes that are set on the ventilator (Chiumello et al., 2014; Pintado et al., 2017; Writing Group for the Alveolar Recruitment for Acute Respiratory Distress Syndrome Trial Investigators et al., 2017; Beitler et al., 2019).

**FIGURE 3:**
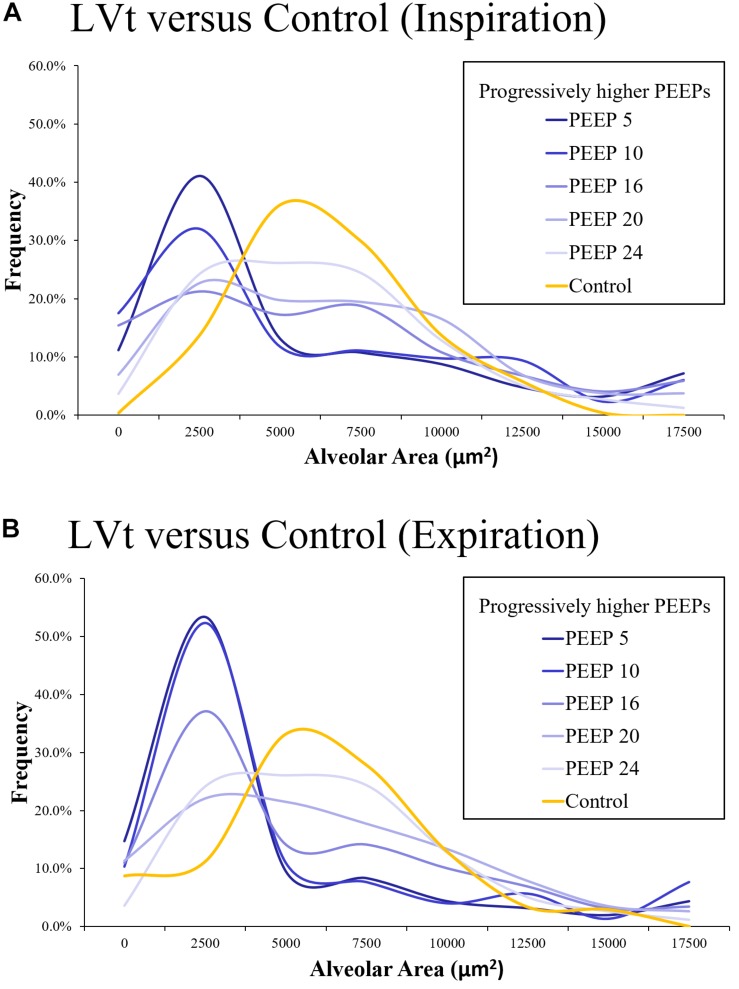
Using *in vivo* microscopy, injured alveoli were ventilated with low tidal volume (LTVV) and evaluated by their response to increasing levels of PEEP and compared against a Control set of alveoli in a healthy, uninjured lung (orange). The distribution of alveolar cross-sectional areas was interpreted with the use of a histogram, with bin sizes selected to be 2500 μm^2^. There is a rightward skew in the injured alveolar cross-sectional areas with a predominant population of small, collapsed alveoli that is more prominent at expiration than inspiration both at inspiration **(A)** and expiration **(B)**. With increasing PEEP, there is a decrease in the skewness and a more normal distribution of alveoli. Even at a PEEP of 24 cmH_2_O, the population of injured alveoli never achieve the same normal distribution as the Control group. With increasing PEEP, it becomes apparent that there are two populations of alveoli: those that remain collapsed despite increases in pressure and those that are responsive to pressure increases, achieving an average alveolar cross-sectional area similar to the Control group (Kollisch-Singule et al., 2016). *Published and color scheme modified with permission, License 4703140582126.*

On a microscopic level using *in vivo* microscopy, injured alveoli were evaluated by their response to increasing levels of PEEP (Kollisch-Singule et al., 2016). Increases in alveolar homogeneity were observed with higher set PEEP and P_aw_, and particularly in the setting of an inverted inspiratory to expiratory ratio (Figure 3) (Kollisch-Singule et al., 2016). Re-establishing alveolar homogeneity becomes more difficult as alveoli become more injured (Kollisch-Singule et al., 2016). Even with PEEP levels set as high as 24 cmH_2_O, the population of injured alveoli never achieved the same normal distribution of alveolar sizes as compared with a Control group of healthy alveoli (Kollisch-Singule et al., 2016). With increasing PEEP, it became apparent that there were two distinct populations of alveoli: those that were responsive to PEEP with a mean cross-sectional area similar to that of the Control group and a group of non-responders with an average cross-sectional area markedly less than that of the Control group (Figure 3) (Kollisch-Singule et al., 2016). This may be one reason why trials that set an arbitrarily higher PEEP do not routinely demonstrate a lower mortality than those patients set with lower PEEP, as the setting adjustments did not target the underlying alveolar pathophysiology (Mercat et al., 2008).

High-frequency oscillatory ventilation (HFOV) is a ventilation mode that should be considered lung-protective with increased mean airway pressures and small tidal volumes promoting recruitment while minimizing lung injury and ventilation-to-perfusion mismatching (Kaczka et al., 2015; Herrmann et al., 2020), but it is generally applied with a single frequency, and has failed to demonstrate improved outcomes over conventional modes (Ferguson et al., 2013; Young et al., 2013). HFOV, like all ventilator modes, relies on inference to predict how changes in pressure, frequency, and flow lead to changes in the alveoli and alveolar ducts (Herrmann et al., 2020). Using a computational model of the human lung, Herrmann et al. (2020) demonstrated that HFOV applied to all lung sizes, but especially larger lung sizes, resulted in regional heterogeneity, and that increasing frequency led to increasing flow heterogeneity. This study therefore demonstrated that increasing frequency in order to lower tidal volume may propagate lung injury by promoting ventilation heterogeneity (Herrmann et al., 2020). Furthermore, these results highlight the importance of considering the individual patient pathology, with each set of lungs benefiting from a different frequency and change in pressure (Herrmann et al., 2020).

One ventilation strategy that has been developed to account for underlying heterogeneity is that of multi-frequency oscillatory ventilation (MFOV) (Kaczka et al., 2015). Herrmann et al. (2018) demonstrated that the use of two frequencies led to reduced acinar strain heterogeneity as compared with the use of either isolated frequency. Kaczka et al. (2015) elevated this idea by proposing MFOV as a method of applying several frequencies simultaneously, thereby taking into account the regional heterogeneity that exists in the premature and injured lung, and minimizing underventilation of one region and overventilation of another. Using a preterm lamb model, MFOV resulted in a more uniform distribution of gas flow to the alveoli with a lower distending pressure, improved oxygenation index, and reduced respiratory system elastance, as compared with traditional single frequency oscillatory ventilation (Kaczka et al., 2015).

In a murine model of lung injury induced by surfactant wash-out and moderate tidal volumes (Vt 12 mL⋅kg^–1^, PEEP 3 cmH_2_O), the lung parenchyma was analyzed with CT (Cereda et al., 2016b). This study demonstrated that ventilator induced lung injury propagated from the induced regions of lung injury concentrically outward (Cereda et al., 2016b). This suggests the alveoli neighboring the injured tissue are subjected to increased strain and subsequent injury from recruitment/derecruitment (Mertens et al., 2009; Cereda et al., 2016b). In a parallel group of rats that received no lung injury but injurious mechanical ventilation by way of large tidal volumes and ZEEP (Vt 30 mL⋅kg^–1^, PEEP 0 cmH_2_O), the ventilator induced lung injury was found to begin in the periphery and spread centrally (Cereda et al., 2016b). This heterogeneous injury pattern is likely due to dependent alveoli/alveolar ducts undergoing recruitment/derecruitment (Cereda et al., 2016b).

In a murine model of surfactant depletion, atelectasis was found to occur primarily in dependent regions of the lung but demonstrated that ventilator induced injury occurred in the non-dependent/non-atelectatic regions (Tsuchida et al., 2006), likely because tidal volumes were distributed to the regions of increased compliance (Gattinoni and Pesenti, 2005). Another murine study of healthy lungs mechanically ventilated with Vt 10 mL⋅kg^–1^ and ZEEP plus recruitment maneuvers used CT and MRI imaging to determine regional lung variations in response to mechanical ventilation (Cereda et al., 2016a). Ventilation with ZEEP led to heterogeneity of and an increase in regional fractional ventilation as measured by MRI, yet did not demonstrate macroscopic atelectasis on CT scan (Cereda et al., 2016a). This study demonstrated both the heterogeneous effect mechanical ventilation may have on the lung and also that subtle alterations in the micro-environment may not manifest on a macro-scale (Cereda et al., 2016a).

## Summary and Conclusion

Investigating the response of alveoli to parameters set on the mechanical ventilator may help to determine protective mechanical ventilation strategies that are also broadly protective at the microscopic level and take the physiology of the various underlying micro-environments into account. The mechanical ventilation modes and settings that appreciate alveolar stability will ultimately be the ones that minimize ventilator induced lung injury and limit the mortality of ARDS. When considering alveolar micromechanics in the acutely injured lung, alveolar instability may be directly related to alveolar heterogeneity and high alveolar Vt.

Alveolar Vt are not determined by the Vt set on the mechanical ventilator but by the number of recruited alveoli available to accommodate that Vt and their heterogeneous mechanical properties. Thus, LVt set on the ventilator can lead to high alveolar Vt when imposed on a partially collapsed lung, but a high Vt set on the ventilator can lead to low alveolar Vt when expressed over a recruited lung with open, homogenous alveoli. In parallel, the pressure that alveoli are exposed to may not be as important as the duration of time those alveoli are exposed to a given pressure. A higher pressure when applied in only brief spurts may not improve long-term alveolar recruitment but may conversely cause harm with large amplitude pressure swings leading to increased strain. But a low pressure exposed over an extended period of time may be insufficient to open alveoli with high opening pressures (Albert et al., 2009). Thus, medium to higher pressures over an extended duration of time may be crucial to achieving alveolar stability. Finally, heterogeneity may be present on several size scales within the lungs, regions of the lung, alveolar subunits, and even the individual alveolar constituents.

CT scans can show regional atelectasis, edema and changes in tissue density to suggest unstable inflation (Cereda et al., 2016a, 2017; Xin et al., 2018) but is not specific enough to detect the immediate response of the alveoli and alveolar ducts to changes in mechanical ventilation (Cereda et al., 2016a, 2019). Electrical impedance tomography has demonstrated usefulness as a potential bedside tool to demonstrate changes in regional ventilation but may not be sensitive enough to detect subtle changes (Kaczka et al., 2015). Oxygenation has been demonstrated to be a poor marker of alveolar instability (Halter et al., 2003; Andrews et al., 2015). Pressure-volume curves have also been shown to be a suboptimal indicator of alveolar recruitment and derecruitment (Dirocco et al., 2007). The ventilator calculates the respiratory system compliance [Vt/(P_Plat_-PEEP)] but does not partition out lung and chest wall compliance (Hess, 2014) where the lung compliance is impacted by alveolar stability (Pavone L. et al., 2007; Knudsen et al., 2018). The lung compliance as a single value may not account for the regional and alveolar heterogeneity associated with the injured lung (Kollisch-Singule et al., 2016), with discordant alveolar compliance even in adjacent alveoli (Perlman et al., 2011; Broche et al., 2017). Thus, many of the bedside maneuvers clinicians have available to guide mechanical ventilation alterations are poor mirrors of alveolar health and stability. With experimental data, this review highlights some of the known mechanical ventilation adjustments that are helpful or harmful at the level of the alveolus.

## Author Contributions

MK-S drafted the manuscript. JS, SB, PA, LG, GN, and NH critically revised the manuscript. All authors read and approved the final manuscript.

## Conflict of Interest

MK-S, JS, PA, GN, and NH have lectured for Intensive Care On-line Network, Inc. (ICON). NH is the founder of ICON, of which PA is an employee. The authors maintain that industry had no role in the design and conduct of the study; the collection, management, analysis, or interpretation of the data; nor the preparation, review, or approval of the manuscript.
